# Supplementary material for: Inter-Oligomer Interaction Influence on Photoluminescence in Cis-Polyacetylene Semiconductor Materials
Source: Polymers (Basel). 2024 Jul 2;16(13):1896. doi: 10.3390/polym16131896 (PMC11244262; doi:10.3390/polym16131896)
Supplement: Supplementary file 1 [file polymers-16-01896-s001.zip › polymers-3035558-supplementary.pdf]

polymers

MDPI

Supplementary Materials

Inter-Oligomer Interaction Influence on Photoluminescence in Cis-Polyacetylene Semiconductor Materials

Kamrun N. Keya [1], Yulun Han [2], Wenjie Xia [1,*] and Dmitri Kilin [2,*]

[1] Department of Aerospace Engineering, Iowa State University, Ames, IA 50011, USA; knkeya@iastate.edu
[2] Department of Chemistry and Biochemistry, North Dakota State University, Fargo, ND 58108, USA; yulun.han@ndsu.edu
* Correspondence: wxia@iastate.edu (W.X.); dmitri.kilin@ndsu.edu (D.K.)

Methods:

To ascertain the total electronic density, the wave functions of the orbitals φ_i^{KS} and their occupation probabilities f_i are integrated as shown below [43,44]:

$$\rho(\vec{r}) = \sum_i f_i \varphi_i^{KS^*}(\vec{r}) \varphi_i^{KS}(\vec{r}) \quad \text{(S1)}$$

This density is crucial in determining the electronic potential field as per the functional derivative of the total energy [43,44]:

$$v[\vec{r}, \rho] = \delta(E^{tot}[\rho] - T[\rho])/\delta\rho \quad \text{(S2)}$$

Our calculations encompass electron interactions with ions and among electrons themselves, including Coulomb, correlation, and exchange interactions as part of the potential energy. This potential is calculated by computing the derivative of the total energy as a function of variations in the electron density across the system. The iterative and self-consistent solution to these equations is facilitated by employing hybrid functionals, notably the HSE06 hybrid functional, which provides a balanced approach between computational efficiency and the accuracy of the exchange-correlation energy calculations [32–34]. The HSE06 functional is parametrized by a screening parameter ω^{-1}, where the short-range Fock exchange contribution is computed as [48–51]:

$$E_{Xc}^{HSE} = aE_X^{HF,SR}(\omega) + (1-a)E_X^{PBE,SR}(\omega) + E_X^{PBE,LR}(\omega) + E_c^{PBE}(\omega) \quad \text{(S3)}$$

Here $E_X^{HF,SR}(\omega)$ refers to the Hartree–Fock exact exchange function component, while $E_X^{PBE,LR}(\omega)$ and $E_X^{PBE,SR}(\omega)$ represent the short-range and long-range components of the PBE (Perdew–Burke–Ernzerhof) exchange function, respectively. The correlation part of the PBE functional is denoted by $E_c^{PBE}(\omega)$. The spinful Kohn–Sham density matrix $\rho\sigma, \sigma'(r, r')$ is integral to the exchange energy calculation, as expressed in the equation [48–51].

$$E_X^{HF,SR}(\omega) = -\frac{1}{2}\sum_{\sigma,\sigma'} \int dr\, dr' \frac{erfc(\omega|r-r'|)}{|r-r'|} \times |\rho\sigma, \sigma'(r, r')|^2 \quad \text{(S4)}$$

Despite the functions equivalence in accuracy to PBE0, the exchange parameter a is typically set to 0.25. The HSE06 functional is fine-tuned to enhance the accuracy, focusing on the screening parameter ω, not a [48–51].

For the electronic density of states (DOS), both total orbital $n(\varepsilon)$ and occupied orbital $n'(\varepsilon)$ contributions are calculated using [45]:

$$n(\varepsilon) = \sum_{i} \delta(\varepsilon - \varepsilon_i) \tag{S5a}$$

$$n'(\varepsilon) = \sum_{i<HO} f_i \delta(\varepsilon - \varepsilon_i) \tag{S6b}$$

Here, the DOS is partitioned into valence and conduction bands, where $i \leq HOMO, for\ f_i \leq \frac{1}{2}$ in the valence band, and $i \geq LUMO, f_i \geq \frac{1}{2}$ for the conduction band. The bandgap is subsequently defined as the energy difference between the LUMO and HOMO levels [54]. In this equation, the Dirac delta function $\delta(\varepsilon - \varepsilon_i)$, pivotal for density of states calculations, is approximated using Lorentzian broadening function [43,44]:

$$\delta(x) = \frac{1}{\pi} \frac{\sigma}{\sigma^2 + x^2} \tag{S6}$$

The broadening parameter σ is set to 0.027 eV to replicate the spectral line width observed in experimental data[61].

Nonadiabatic Calculations: Analysis of Observables

Our method involves delineating changes in the non-equilibrium electron and hole distributions post-photoexcitation, defined mathematically as [45]:

$$\Delta n(\varepsilon, t) = n^{''}(\varepsilon, t) - n'(\varepsilon) \tag{S7}$$

This equation quantitatively captures the population dynamics, with a positive $\Delta n > 0$ indicating an increase (electrons) and a negative $\Delta n < 0$ indicating a decrease (holes) at a given energy level ε. The temporal progression of populations for the highest occupied and lowest unoccupied molecular orbitals, HO and LU, respectively, is governed by the equation [30,45]:

$$P_{e(h)}(t) = 1 - exp\left(\frac{t}{\tau^{e(h)}}\right) \tag{S8}$$

In this context, e and h symbolize electrons in the conduction band and holes in the valence band, respectively, with $\tau^{e(h)}$ representing their mean relaxation times. This parameter is instrumental in characterizing the relaxation kinetics for both charge carriers. We further characterize the expected energy values of these charge carriers using [30,45]:

$$< \Delta\varepsilon_e > (t) = \sum_{i} \rho_{ii}(t) \varepsilon_i(t) \tag{S9}$$

To provide a dimensionless representation of energy, we normalize these values [30,45]:

$$< E_e > (t) = \frac{< \Delta\varepsilon_e > (t) - < \Delta\varepsilon_e > (\infty)}{< \Delta\varepsilon_e > (0) - < \Delta\varepsilon_e > (\infty)} \tag{S10}$$

When modeled as a single exponential, this relationship implies an energy dissipation model [30,45]:

$$< E_e > (t) = exp\{-k_e t\} \tag{S11}$$

These equations enable us to elucidate the relaxation processes and the time-dependent behavior of the excited states in our system.

Supplemental Figures

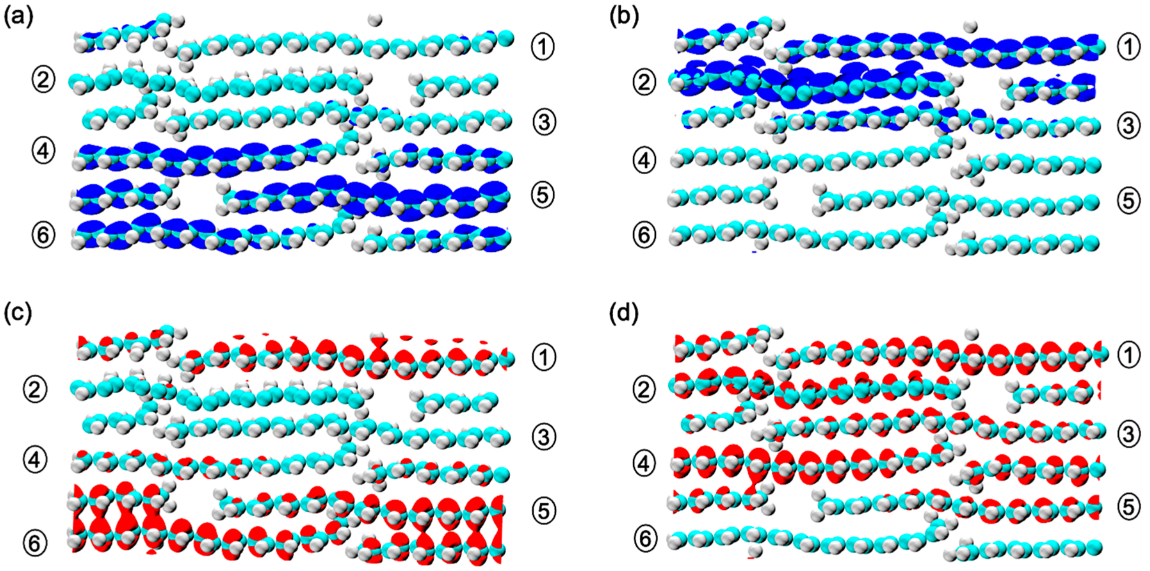

Figure S1. Visualization of Charge Densities in cis-PA Molecular Orbitals. Charge densities of the frontier molecular orbitals for an undoped cis-polyacetylene (cis-PA) model in the ground state are depicted, with computations performed using HSE06 hybrid functionals. Panel (a) portrays the HO-1 orbital, represented by state 471. Panel (b) exhibits the model's HO orbital, state 472, in its neutral form. Panel (c) shows the LU orbital, state 473, and panel (d) illustrates the LU+1 orbital. The visualized iso-surfaces are generated with an iso-value of 0.0028 to delineate regions of significant electron concentration.

A detailed examination of Figure S1 reveals the distribution of charge across ensemble oligomers. Specifically, the Highest Occupied (HO) molecular orbital extends across the first and second oligomers, while the Lowest Unoccupied (LU) molecular orbital spans the first, fifth, and sixth oligomers, indicating signs of hybridization. Initially, hole populations are predominantly found in the fourth oligomer. At the same starting point, electron density is primarily seen in the second and third oligomers. Over time, this electron density transitions towards the fifth and sixth oligomers, as documented in Figures S3 and S4. Conversely, hole density migrates from the third to the second oligomer. Notably, the HO orbital 472 exhibits a pronounced charge density mainly in the second oligomer, whereas the LU orbital 473 has its greatest localization in the sixth oligomer, with significant density also observed in the fifth.

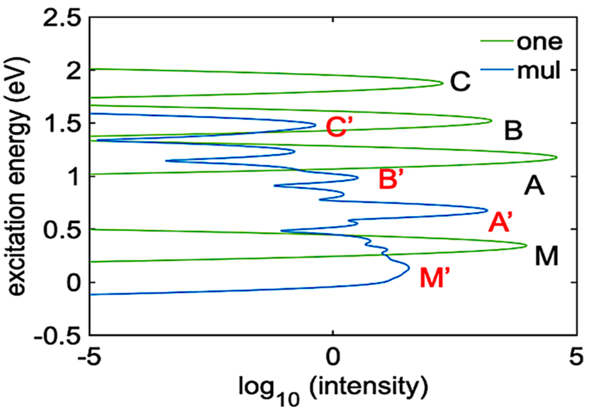

Figure S2. Time-Integrated PL Spectral Analysis for cis-PA Oligomers. Presented here are the time-integrated photoluminescence (PL) results, contrasting single cis-PA oligomer spectra (in green) with those of cis-PA oligomer ensembles (in red). The inter-band transitions, designated as A/A', B/B', and C/C', manifest in the spectra of both single and multiple oligomer systems. However, the

intra-band transition marked as M appears solely in the PL spectrum of the individual oligomer, while the corresponding transition M' is unique to the PL spectra of the oligomer ensembles. 'One' indicates a single oligomer, and 'mul' references the oligomer ensemble within the cis-PA system.

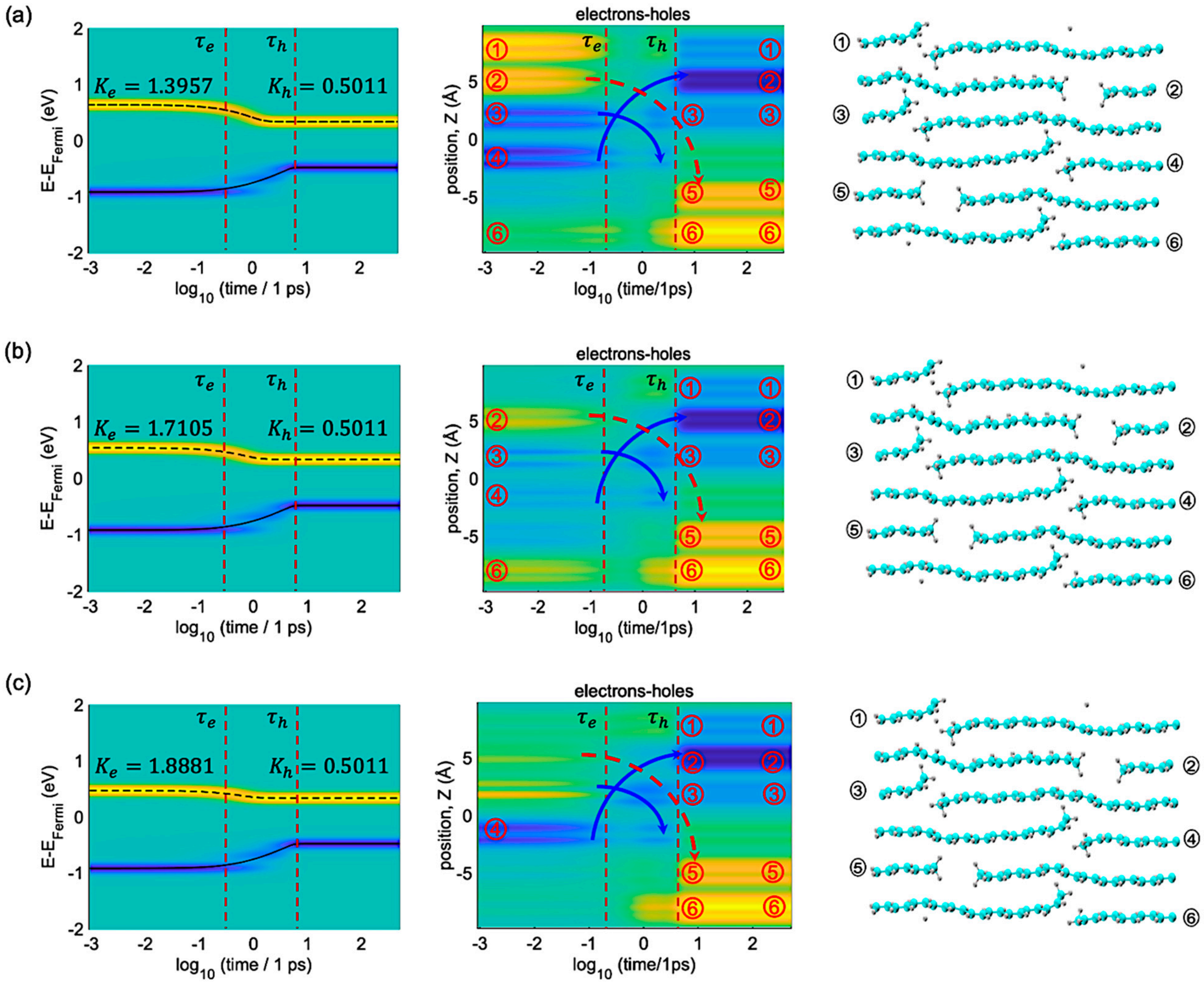

Figure S3. Dynamics of charge carriers in cis-PA ensemble of oligomers for different initial conditions. This figure displays the dynamics of charge carriers in cis-PA oligomer ensembles following photoexcitation, focusing on three transitions with notable oscillator strengths: (a) from HO-6 → LU+5, (b) from HO-6 → LU+3, and (c) from HO-6 → LU+2. The progression of charge across energy and time is depicted in the left panels. Expectation values for energy within the conduction and valence bands are plotted as dashed and solid lines within an energy distribution framework. Central panels illustrate charge density's spatial and temporal evolution, with electrons, the equilibrium state, and holes colored in yellow, green, and blue, respectively. The discrepancy between the solid and dashed lines indicates the electric dipole moment. The periodicity of the oligomer assembly is visualized in the right panels, providing a structural context for the ensemble of cis-PA oligomers.

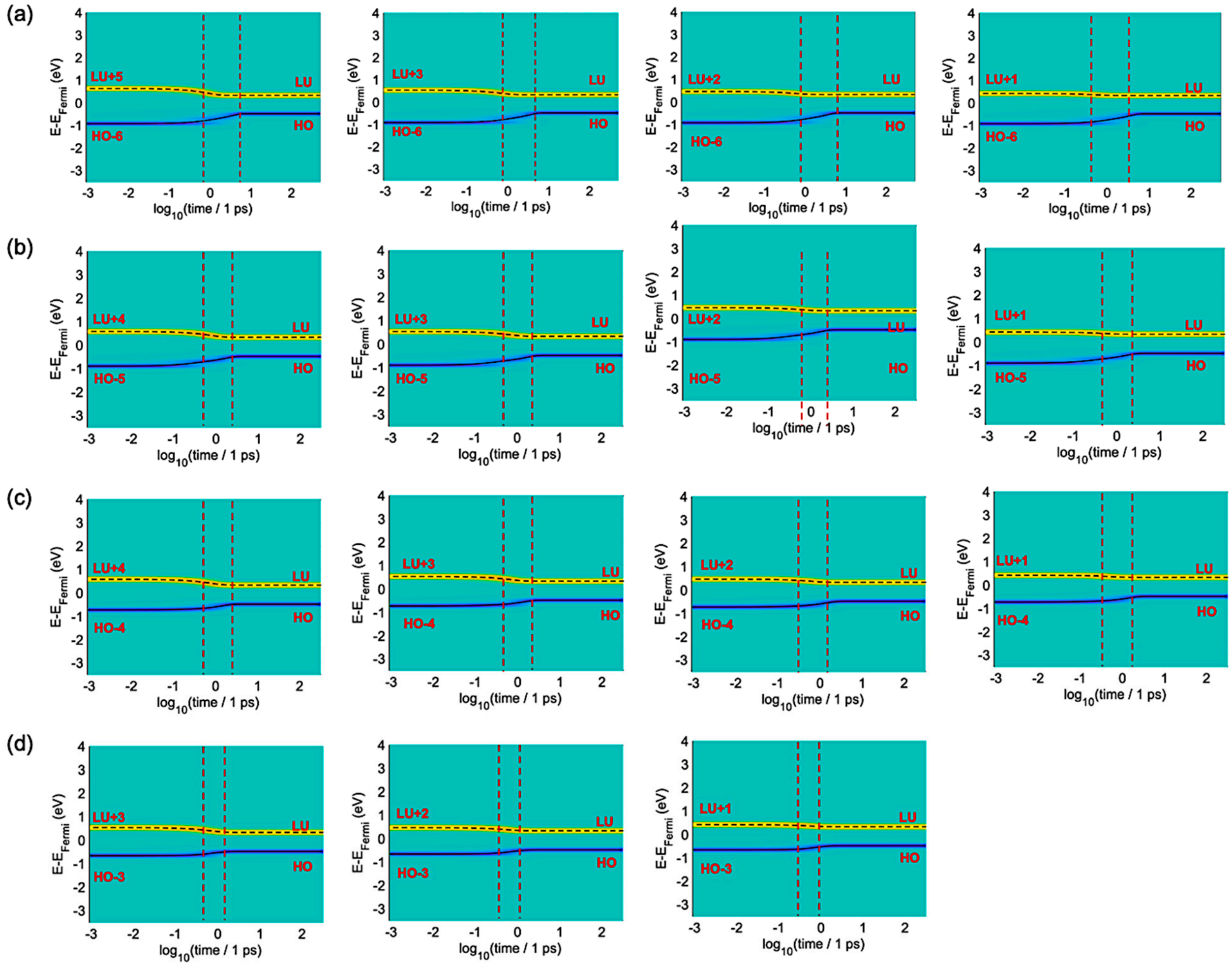

Figure S4. Dynamics of charge carriers in the ensemble of oligomers photoexcitation for different representative initial transitions possess higher oscillator strength.

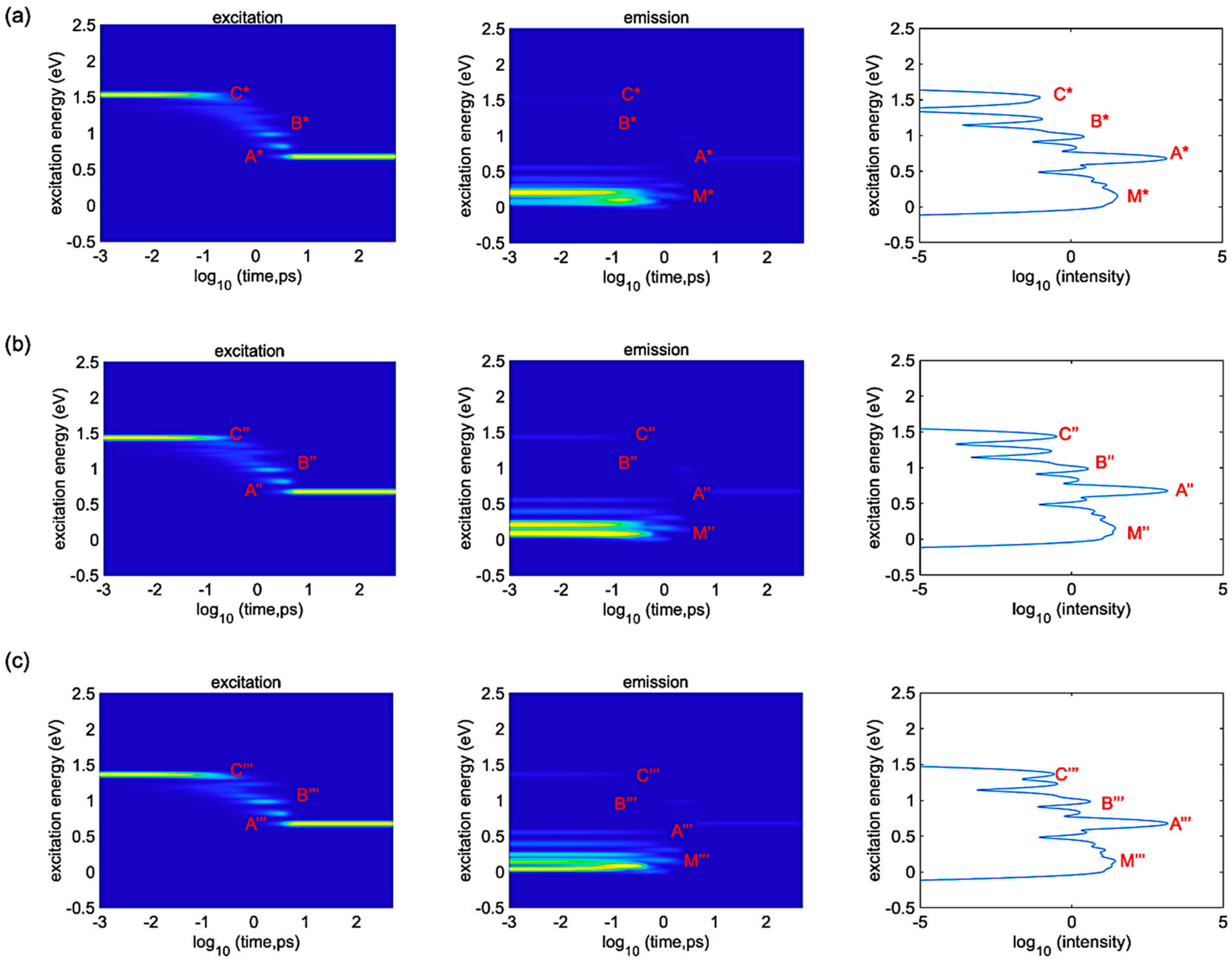

Figure S5. Dynamics and emission spectral analysis of photoexcited cis-PA oligomer ensembles. This figure delineates the photoexcitation dynamics of cis-PA oligomer ensembles at an excitation energy of 1.5 eV. The left panel visualizes the energy dissipation of excitons over time, with a color gradient from yellow, indicating peak population, to navy blue, representing no population. The middle panel depicts the time-resolved emission spectrum, with color intensity reflecting the transition's oscillator strength, ranging from maximum (yellow) to none (navy blue). The right panel presents the integrated emission spectrum, highlighting features *A′*, *B′*, and *C′* for inter-band transitions and M′ for an intra-band transition, with transitions (a) from *HO-6* → *LU+5*, (b) from *HO-6* → *LU+3*, and (c) from *HO-6* → *LU+2*.

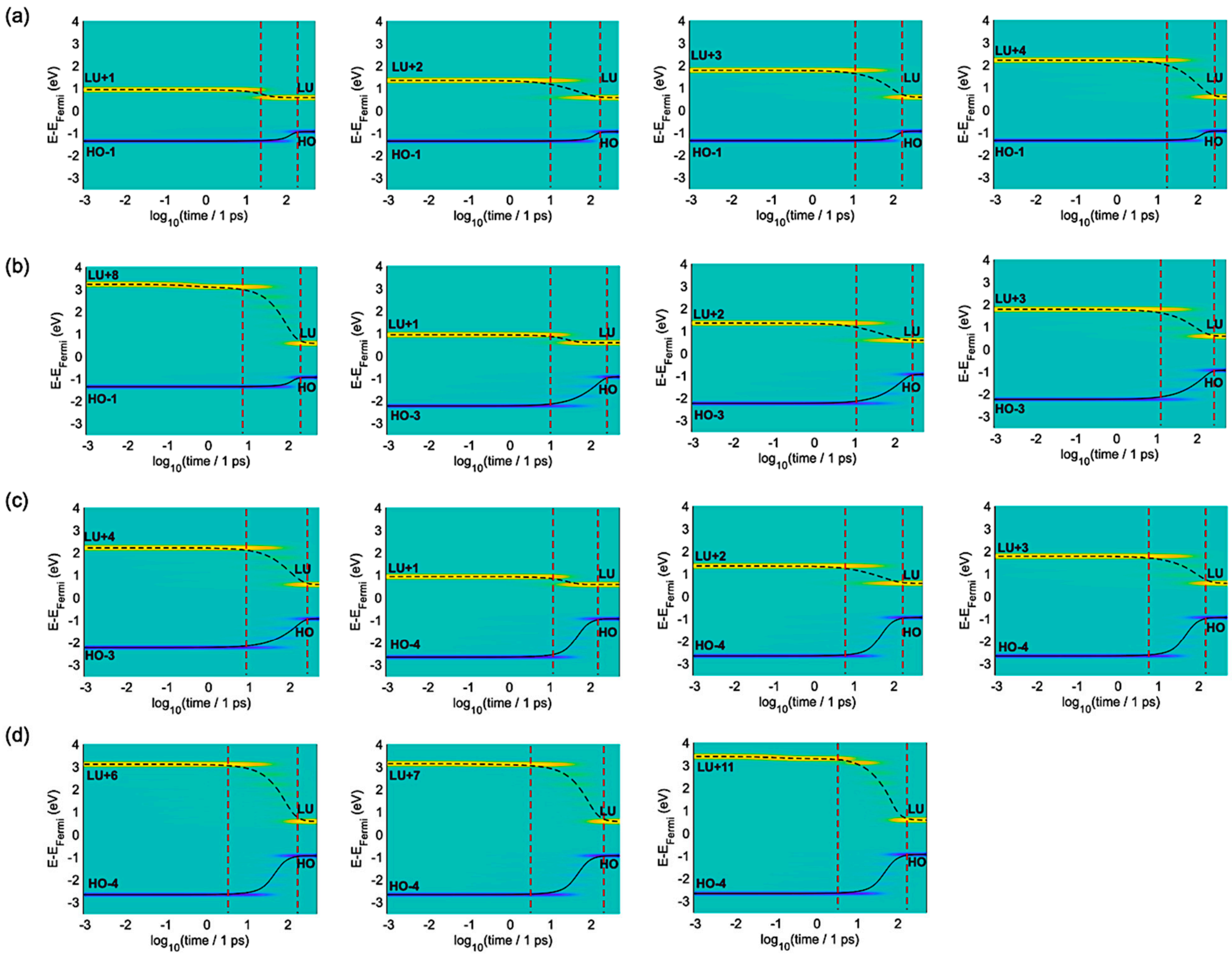

Figure S6. Dynamics of charge carriers in the single oligomer photoexcitation for different representative initial transitions possess higher oscillator strength.

Reference

61. Barcaro, G.; Broyer, M.; Durante, N.; Fortunelli, A.; Stener, M. Alloying Effects on the Optical Properties of Ag–Au Nanoclusters from TDDFT Calculations. *J. Phy. Chem. C* **2011**, *115*, 24085–24091. https://doi.org/10.1021/jp2087219.

Disclaimer/Publisher's Note: The statements, opinions and data contained in all publications are solely those of the individual author(s) and contributor(s) and not of MDPI and/or the editor(s). MDPI and/or the editor(s) disclaim responsibility for any injury to people or property resulting from any ideas, methods, instructions or products referred to in the content.
